# What Do You Mean by That?! An Electrophysiological Study of Emotional and Attitudinal Prosody

**DOI:** 10.1371/journal.pone.0132947

**Published:** 2015-07-15

**Authors:** Steven Wickens, Conrad Perry

**Affiliations:** Swinburne University of Technology, Hawthorn, Australia; The University of Nottingham, UNITED KINGDOM

## Abstract

The use of prosody during verbal communication is pervasive in everyday language and whilst there is a wealth of research examining the prosodic processing of emotional information, much less is known about the prosodic processing of attitudinal information. The current study investigated the online neural processes underlying the prosodic processing of non-verbal emotional and attitudinal components of speech via the analysis of event-related brain potentials related to the processing of anger and sarcasm. To examine these, sentences with prosodic expectancy violations created by cross-splicing a prosodically neutral head (*‘he has’*) and a prosodically neutral, angry, or sarcastic ending (e.g., *‘a serious face’*) were used. Task demands were also manipulated, with participants in one experiment performing prosodic classification and participants in another performing probe-verification. Overall, whilst minor differences were found across the tasks, the results suggest that angry and sarcastic prosodic expectancy violations follow a similar processing time-course underpinned by similar neural resources.

## Introduction

The ability to evaluate and integrate verbal and non-verbal speech cues is integral to successful human communication. Moreover, non-verbal speech cues possess much functional utility, which includes communicating the intention, behaviour, and affective state of a speaker, as well as providing linguistic and syntactic utility (e.g., [1]). While these varying roles possess overlap in particular instances, different types of prosody can be conceptualised as functionally distinct and have been more formally classified in relation to emotional, attitudinal, linguistic, and inarticulate characteristics [2].

Affective prosody is comprised of both emotional and attitudinal components, although research has tended to focus on the former [2]. Emotional prosody relates to the expression of emotion by tone of voice and typically conveys a speaker’s emotional state (e.g., anger, happiness–‘I am happy’). In contrast, attitudinal prosody (e.g., [3]) relates to the communication of information associated with a speaker’s attitude that may alter the meaning of an utterance. In general, it has been conceptualized with respect to a speaker’s behavior (e.g., ‘*I am being sarcastic’*).

Given the limited number of prosodic elements (i.e., pitch, duration, rhythm etc.), emotional and attitudinal prosody rely on overlapping acoustic cues [4]. Although modulation of pitch is important for the expression of both types of prosody, emotional expressions rely more heavily on particular parameters such as voice quality (i.e., shrillness, harshness, softness etc.), whereas prosodic contour (i.e., pitch and rhythm modulation) is vital to attitudinal expressions [5]. Importantly, people can distinguish between different types of attitudes and emotions based on these prosodic characteristics [6–8]. In addition, the extent to which different cues contribute to the recognition of different types of expressions is likely to differ, and the level of consensus as to which cues are vital for the identification of expressions is dependent on the attitudes and/or emotions examined. For example, in relation to the expression of anger in English, there is consistency across studies in terms of the pitch speakers use, with almost all suggesting that angry sentences are conveyed with a higher pitch than neutral ones (e.g., [9–12]). Alternatively, there is less agreement about which prosodic cues consistently differ compared to neutral sentences with verbal sarcasm. For example, Rockwell [13] provided evidence to suggest that sarcastic speech uses a slower tempo, lower pitch, and greater intensity than speech with neutral valence. Bryant and Fox Tree [14], alternatively, found that sarcasm uses a higher pitch and that the duration of their neutral and sarcastic utterances did not significantly differ. Moreover, the authors suggested that amplitude variability and global processing might be important. Apart from sentences with some form of non-neutral valence, speech of a neutral valence may also differ across language dialects, thus making comparisons between neutral and non-neutral speech even more complicated. For example, younger speakers of New Zealand and Australian English often significantly raise the pitch of neutral sentences towards their end [15], and even then display variability. As such, the overall pitch and pitch variability of neutral sentences would increase compared to other English dialects and thus dilute any differences with other types of prosody on these measures.

### The Neural Basis of Affective and Attitudinal Processing

In general, research has supported a right hemisphere dominant process for prosodic processing, in contrast to the verbal-linguistic dominant functionality of the left hemisphere (e.g., [2]). More specifically, the right hemisphere specialization hypothesis is typically in reference to emotional prosody, whereby converging evidence from lesion studies has revealed degradation of emotional prosody comprehension in patients with right hemisphere damage [16]. Alternatively, neuroimaging data suggests that both the left and right hemispheres are important for processing emotional prosody, and that the left hemisphere may be more involved with increased task demands [17,18].

One explanation of these findings is via the cue-dependent hypothesis, which suggests that the lateralization of prosodic processing is dependent on the acoustic cues that are critical for the extraction of meaning and that these cues are processed in different anatomical areas of the brain [19]. More precisely, it has been suggested that the two hemispheres process information on different temporal scales, with the left hemisphere using a finer temporal scale than the right hemisphere [20]. Accordingly, the left hemisphere should be more efficient in the analysis of rapidly changing information in speech, such as durational cues. Alternatively, the right hemisphere, which functions with a lower temporal resolution, should be more efficient at processing information related to pitch [21]. Based on these differences, emotional expressions, which tend to involve the modulation of pitch [22], are more typically associated with acoustic cues processed in the right hemisphere.

Consistent with the cue-dependent hypothesis, Schirmer and Kotz [23] propose a three-stage model of vocal emotional processing. In the model, the role of the auditory cortex is initially implicated in the modulation of early sensory acoustic information for which the right hemisphere is dominant. Subsequently, the emotional significance of vocalizations is processed with respect to the different patterns of emotionally salient acoustic cues, proposed to occur within the anterior superior temporal sulcus (STS) for which laterality is congruent with the cue-dependent hypothesis. Emotional information is then available for higher order cognitive processes, such as evaluative judgments, which are subsequently mediated by the right inferior and orbitofrontal cortex.

The first two stages of the Schirmer and Kotz model have also been suggested to be those used in attitudinal processing by Mitchell and Ross [2]. However after a comprehensive review of neuroimaging and lesion data, Mitchell and Ross suggested that attitudinal processing deviates from simple emotional processing (e.g., anger) in the third stage activation of frontal regions. In particular, they suggested that the first two stages were used for the simple decoding of auditory cues and used the same neural processes, irrespective of the actual type of prosody being processed. Alternatively, with the third stage, they suggested that attitudes are likely to be similar to complex emotions and require significantly higher levels of processing than simple emotions. They also noted that, empirically, the prosody of complex emotions had already been shown to activate medial prefrontal areas more than simple emotions, suggesting that they require a number of additional types of processing to occur. Examples given by the authors of instances where attitudes would demand a higher level of processing than simple emotions included judging the authenticity of an utterance, processing to do with theory of mind, and interpreting social intentions.

### The Present Study

Event related potentials (ERPs) have been extensively used to examine the online temporal patterns and neural correlates associated with various types of sentence level prosodic processing (e.g., [24–31]). A number of different paradigms have been used and a large number of components have been found to be associated with emotional processing and to a lesser extent attitudinal processing. A summary of the paradigms and results found from some of these studies appears in Table 1. As can be seen, some of the results appear to be quite different from each other. In particular, the results of Liu et al. [32] and Paulmann and Kotz [33] demonstrate the opposite results entirely. Similarly, the cortical distribution of effects found in Kotz and Paulmann [27] and Chen et al. [25] differ despite the use of the same paradigm. One explanation of such differences may be that some components are relatively sensitive to task/stimuli differences. For example, in the study of Paulmann and Kotz, the prosody of words in sentences was examined whereas Liu et al. used affectively valanced vocalizations (e.g., sighing). Likewise, in the study of Kotz and Paulmann, German stimuli were used whereas Chen et al. used Mandarin ones, and because Mandarin is a tonal language and German is not, the way different areas of the brain are recruited due to tone/intonation interactions may differ (e.g., [34]). Additionally, Chen et al. noted that there are important differences in the acoustic characteristics Chinese speakers use to recognize emotion compared to English speakers.

**Table 1:** Overview of ERP studies on Emotional and Attitudinal Prosody.

Study	Paradigm/Stimuli	Type of Prosody	Task/Experiment	Findings
Schirmer et al. [31]	Syllables (Non-words)	Angry/Neutral	Oddball Paradigm: Syllable judgment	Mismatch Negativity (200ms) for emotional deviations with a right anterior distribution–restricted to women.
Paulmann and Kotz [33]	Simple sentences	Angry/Fear/Disgust/Happy/Surprise/Sad/Neutral	Probe-verification	Decreased P200 amplitude for angry prosodic sentences relative to prosodically neutral sentences.
Liu et al. [32]	Non-verbal utterances (human + monkey)	Angry/Happy/Neutral	Human/Monkey voice discrimination	Increased P200 amplitude for angry prosodic sentences relative to prosodically neutral sentences.
Kotz and Paulmann [27]	Cross-spliced sentences (neutral head, emotional body)	Angry/Happy/Neutral	Exp 1: Probe-verification. Exp 2: Prosodic Categorization	Prosodic Expectancy Positivity (PEP) effect (~350ms) for emotional violations with a whole head distribution for Exp 1 and a right anterior lateralized distribution for Exp 2.
Chen et al. [25]	Cross-spliced sentences (neutral head, emotional body)	Angry/Neutral	Exp 1: Probe-verification. Exp 2: Prosodic Categorization. Exp 3: Use of Spectrally Rotated Counterparts as Controls	A bilateral anterior maximal early negativity (150-250ms) and positivity (250-400ms) for emotional violations, independent of task demands and greater than for spectrally rotated counterparts. A late centro-parietal positive complex (450-900ms) was seen only for task relevant conditions.
Paulmann et al. [29]	Cross-spliced sentences (neutral head, emotional body)	Angry/Questioning/Neutral	Prosodic Categorization	Emotional prosodic violations elicited a posterior positivity (~470ms); linguistic prosodic violations elicited an anterior positivity (~620ms); and combined linguistic and emotional violations produced an early positivity (~170ms) with a more broad distribution.
Regel et al. [37]	Discourse Context	Irony/Literal	Prosodic Categorization	A bilateral posterior P600 effect for ironic relative to literal sentences, independent of target sentence presentation mode (i.e. auditory or visual). Early divergence from literal sentences showed a left lateralized anterior negativity for prosodic irony (~250ms) while a P200 effect was found for visual irony.
Rigoulot et al. [38]	Question–Answer Context	Insincere/Sincere	Prosodic Categorization	An anterior right lateralized P600 effect for insincere compared to sincere intonations.

*Note*. Cross-spliced sentences = Prosodic expectancy paradigm; Probe-verification = attention directed away from prosody via word identification; Prosodic categorization = attention directed towards prosody.

Alternatively, another possibility for the conflicting results is that due to the small number of studies examining these effects, more minor differences may simply be due to unfortunate statistical anomalies. These may have occurred in part because many of the studies have used relatively low numbers of items per cell for ERP studies (e.g., [35]), which is likely a consequence of the time it takes to present stimuli. For example, some studies have used 30 items per cell [33] and around 50 is common (e.g., [29]). Given this, the extent to which different effects can be replicated and under what conditions are important considerations.

The present study investigates the time-course underlying the on-line processing of emotional and attitudinal prosody using ERPs to expand the understanding of how prosodic information is processed. As such, a primary aim of the study is to bridge the gap in the literature pertaining to processing differences between emotional and attitudinal prosody. In terms of emotional prosody, ERP effects have demonstrated consistency across a range of emotions [28], with anger being the emotion most commonly used [25,27,29]. Anger also has well known acoustic properties [11,25,36]. By adopting anger as the representative of emotional prosody, the current study will allow comparison to previous research. In terms of attitudinal prosody, sarcasm will be employed even though other types of attitudinal prosody have been previously investigated (General irony [37]; Sincerity [38]). Sarcasm was chosen primarily due to it containing a negative aspect [39] like anger, meaning that both types of stimuli evoke negative valence. Importantly, however, the perceived impact and valence of sarcasm can differ to anger in certain contexts (e.g., [40,41]), such as when it is used in humor. This may either increase the negative aspect to the victim (e.g., further humiliation which may be amusing to others who are not the victim), or lessen the negative overall aspect when it is done as a form of politeness (e.g., making fun of an accidental faux pas). Thus, whilst anger and sarcasm may share some negative aspects, this similarity can often be context dependent. As such, matching on the negative valence has its limits. In addition, as the current study employs no situational context, the interpretations of the expressions are likely to be more dependent on the individual’s perception of the acoustic parameters. As a result, there is a trade-off for external validity. Another key problem with examining sarcasm is that it is less clear what the acoustic correlates are (see e.g., [42–44] and further discussion above) and that some forms may require the combination of prosodic and pragmatic/contextual features [3,45]. Neither of these problems is insurmountable since stimuli can simply be generated a-priori so that they are clearly recognised as sarcastic, even if this translates to a limited range of sarcastic expression.

The paradigm adopted will be the prosodic expectancy violation paradigm [25,27,29]. Stimuli consist of cross-spliced/merged syntactically matching and semantically neutral sentences with a prosodically neutral head (‘*he has’*/*’she has’*) and a neutral, angry, or sarcastic prosodic ending (e.g., ‘*a serious face’*). The basic premise is that upon participants hearing the prosodically neutral head, an expectation is generated that the same prosody will continue as the sentence unfolds [27]. A violation of this expectancy will subsequently occur when the cross-spliced emotional/attitudinal prosodic endings are presented. Alternatively, sentences with a neutral prosodic ending do not cause a violation of expectancy and therefore constitute the control condition. Similar to Kotz and Paulmann [27] and Chen et al. [25], the current study also manipulates task demands via introducing two variations of the same experimental design: a prosodic-categorization task and a probe-verification task (Experiment one and two, respectively). The purpose of this manipulation is to influence the extent to which attention is directed towards prosodic processing, thereby permitting a means to assess and compare the automaticity of processing and the effects of task demands on the two types of prosody.

Cross-spliced sentences were used in this study rather than simple sentences as we were interested in examining the extent to which angry and sarcastic prosody differ in terms of their temporal processing. In particular, one manner that emotional stimuli might be prioritized for more immediate processing is via their acoustic differences compared to normal speech. In turn, this could lead such utterances to differ from prosodically attitudinal ones in two important ways. First, as noted above, the acoustic correlates of sarcasm may be more variable than anger, and this may result in a slower detection of prosodic changes than with anger. Second, the cues may be potentially less emotionally relevant than anger–that is, their functional context may differ [27] because the reception of anger may signal the need for an immediate response due to it being more commonly associated with adaptive factors such as violence compared to sarcasm; the latter of which may reflect more subtle higher order social functionality. Thus, it may be more adaptive for people to be attuned to prosodic cues associated with anger than sarcasm. Obviously, cross-spliced sentences are more artificial than naturally occurring sentences, and important prosodic information from the head of sentences [46] may be lost. However, the sentences used in the current study had a neutral head and then a potentially sarcastic or angry body which is a type of change that is relatively common in written form where the speech of a character is in quotes or implied, albeit in a different voice.

There were three main aims of this research. First, to replicate previous findings relating to the temporal processing of emotional prosody in the context of the prosodic expectancy paradigm [25,27–29]. Based on the results of Chen et al. [25], it is expected that differences between angry and neutral stimuli may modulate a very early negative component in the 100-200ms time window. It is also expected that emotional violations will elicit a significant positive deflection in the 200-350ms time window resembling a prosodic expectancy positivity (PEP effect) [25,27–29]. Finally, when attention is directed towards prosodic processing, a late positive complex may be seen for emotional prosody that is indicative of online integration processes [25].

Second, it is less clear what components are likely to be associated with sarcastic processing. Given that the time-course of sarcastic prosody has not yet been examined within this paradigm, only tentative suggestions can be made. The most likely finding is that prosodic violations may deviate from matching neutral sentences due to early sensory change-detection processes (i.e., an early negative effect). In terms of later components, as attitudes differ from emotions [2], possessing less salient acoustic cue configurations and reflecting higher socially derived affectively charged dispositions, attitudinal violations may be processed later in time compared to emotional violations and hence cause a later PEP effect compared to emotional prosody. This expectation is derived in part from the finding that emotional prosodic violations were found to generate earlier PEP effects compared to linguistic prosodic violations [29].

Finally, the extent to which automaticity and task demands affect processing will be examined. For emotional prosody, previous research has shown that the presence of prosodic processing and the temporal PEP effect pattern is relatively independent of task demands [27]. Alternatively, attitudinal prosody is argued to demand a level of selective attention [3], and thus if automaticity effects emerge across the two experiments, the difference in the amplitude of the PEP effect is likely to be greater for attitudinal compared to emotional prosody.

## Experiment One

In this experiment, the prosodic expectancy paradigm was used to examine the processing of anger and sarcasm when the task forced participants to process the prosody of the sentences.

### Method

#### Participants

Fifteen healthy individuals (9 males, 6 females), aged 18–25 years (*M =* 22.13; *SD* = 1.77), participated in Experiment one. All were native Australian English speakers with normal or corrected-to-normal vision, without any known psychiatric or neuropsychological disorder, and were not currently taking any psychiatric drugs or substances, as provided by self-report. All participants provided informed written consent and were compensated for their participation. This experiment was approved by the ethics committee of Swinburne University of Technology.

#### Stimulus Material

In accordance with the prosodic expectancy paradigm used in Kotz and Paulmann [27], sentences were created comprising a common head structure (*‘he/she has’*) and a body structure (i.e. ‘*a serious face*’). The latter body structures were generated from 150 syntactically matching and semantically neutral sentences comprised of three words beginning with either an ‘*a*’ or ‘*an*’ determiner. To create the critical experimental manipulations, each sentence body was recorded in three different intonations: i) emotional (angry tone), ii) attitudinal (sarcastic tone), and iii) neutral. In addition, 100 filler and 10 practice sentence bodies of a similar syntax and length were created and recorded. The filler sentences all had a neutral tone.

All stimuli were produced by the first author (a native Australian English speaker who spoke with an accent from the same region as the participants) and recorded in a recording studio using a Rode NT2-A microphone with a pop filter at a sampling rate of 48 kHz. The audio recordings were recorded and edited using Protools 9 software. Importantly, given that the stimuli were not accompanied by contextual cues to facilitate interpretation, only those stimuli that were a-priori determined to be easy to prosodically categorize by the authors and a number of collaborators were used. This included 9 observers who categorized the final set of stimuli. They showed accuracy rates of 99.1%, 98.4%, and 98.0% on average when classifying the angry, sarcastic stimuli, and neutral stimuli respectively, and 98.7% on the fillers. Unfortunately, due to a computer problem, we did not collect this data on the actual participants, although given all of the observers were at ceiling it would be very unlikely that the participants in the EEG study would have had high error rates.

**Fig 1:**
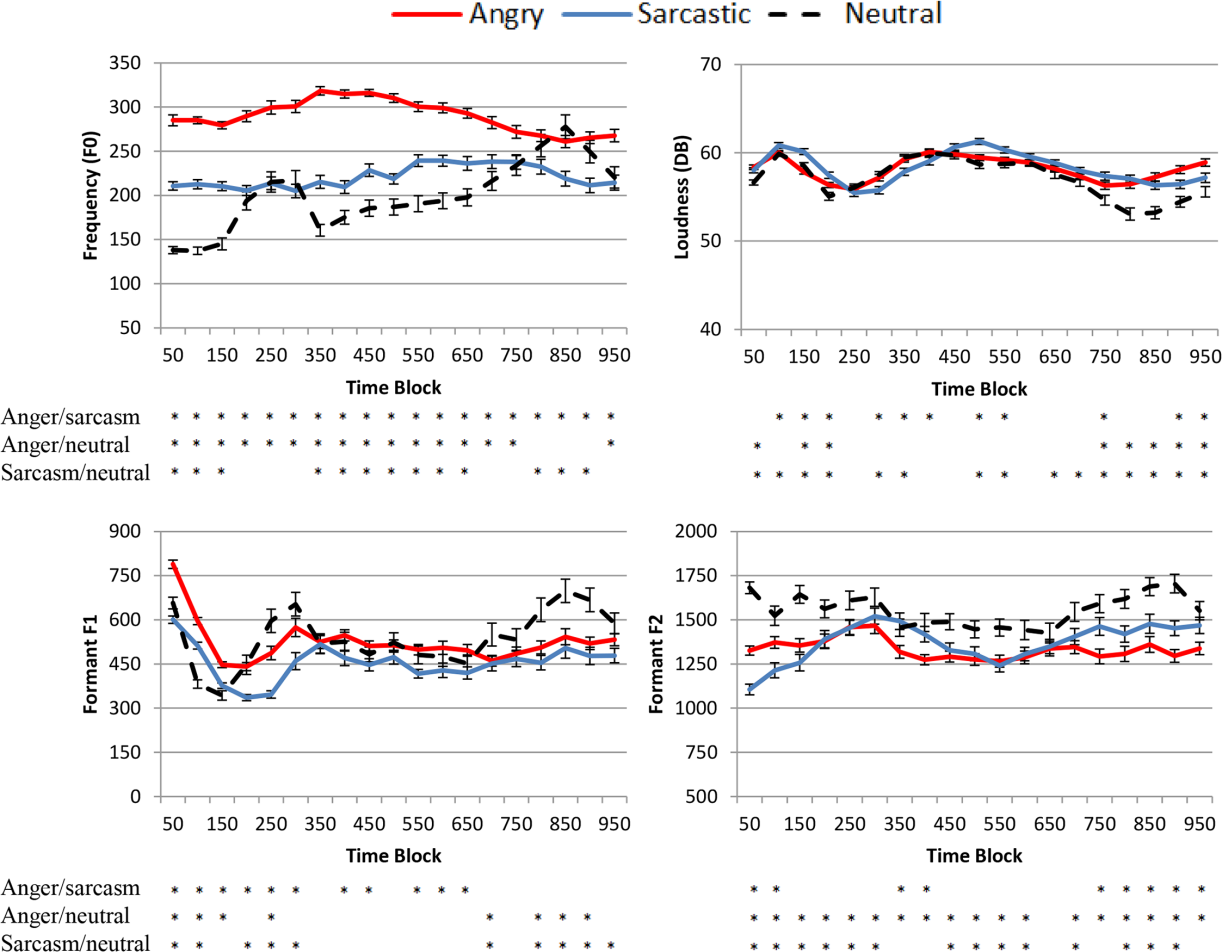
Mean fundamental frequency F0 (Hz), Loudness (DB), Formant F1 (Hz), and Formant F2 (Hz) values for the first 950ms of the angry, sarcastic, and neutral sentences in 50ms bins, starting from 0-50ms. Error bars are +/- 2 Standard Errors. The asterisks under the graphs represent whether there was a significant difference between the conditions at each bin at an uncorrected p < .05 level.

Final cross-spliced versions of the sentences were created by pseudo-randomly assigning a head structure to each sentence body. This resulted in three types of stimuli: i) prosodically neutral matching target sentences, ii) prosodically mismatching target sentences (angry or sarcastic prosodic violations), and iii) prosodically neutral filler sentences. In total, there were 450 critical stimuli sentences (150 for each of the three prosodic categories used), which were evenly split into three counterbalanced groups, with each group also having the 100 fillers and 10 practice sentences. The fillers and practice sentences were structured identically to the critical sentences, that is, a head was attached to a body structure. Thus, the most common type of sentence in each group had a prosodically neutral body (150 sentences vs. 50 with sarcasm and 50 with anger). To examine the linguistic characteristics of the sentences that were generated, the materials were acoustically analysed using the Praat [47] and Matlab v2014a software for fundamental frequency (F0), formant F1 and F2, and intensity (decibel). Apart from overall duration, this was done by dividing the sentence bodies into 50ms bins and taking the average of these values for the first 950ms. This latter value was used because all of the sentence bodies were over this duration. The results appear in Fig 1.

In terms of the overall duration, although a significant difference was found (duration (*F*(1.81, 283.63) = 60.71, *p* <. 001, *partial η^2^* = .28), the absolute mean durations were relatively similar (In seconds, Angry: 1.53; Sarcastic: 1.60; Neutral: 1.53). The results from the analysis of the bins, alternatively, showed that there were large differences on the measures examined, and that these occurred from the beginning of the sentence bodies. Most notably, anger had a higher pitch than the neutral and sarcastic stimuli, apart from at late time intervals where the pitch of the neutral utterances increased, and this result was especially strong (for example, the first 10 time bins showed *p* values less than 10^−8^ in both comparisons). This rise towards the end of neutral sentences is a common pattern in the speech of younger Australians in the geographical location the study was run. Sarcasm also displayed a higher pitch than the neutral utterances, although the difference was not as large as anger. Apart from difference in pitch, the angry speech also displayed heightened intensity, which is consistent with other English studies, although the differences were relatively small (albeit significant) [42,44], which was at least in part due to a deliberate effort made in recording to keep this measure similar across stimuli types. There were also clear differences between the different sentences at early time windows on Formant F1 and F2. Taken together, all of the measures suggest that, with our stimuli, anger, sarcasm, and neutral intonations are clearly prosodically different from each other on easily quantifiable measures from early time points.

In addition to the critical stimuli, the filler sentences were compared with the neutral critical sentences using the same four measures. The results showed that there were only minor differences in the values at the different times bins across the four measures, with 17 values under a *p* < .05 criterion and 8 under a *p* < .01 criterion. The pattern across the measures also appeared very similar and the classification of the stimuli as neutral in our validation phase was at ceiling. It therefore seems reasonable to suggest that, for all intents and purposes, the filler sentences worked as sentences with neutral valence.

#### Stimulus Presentation

**Fig 2:**
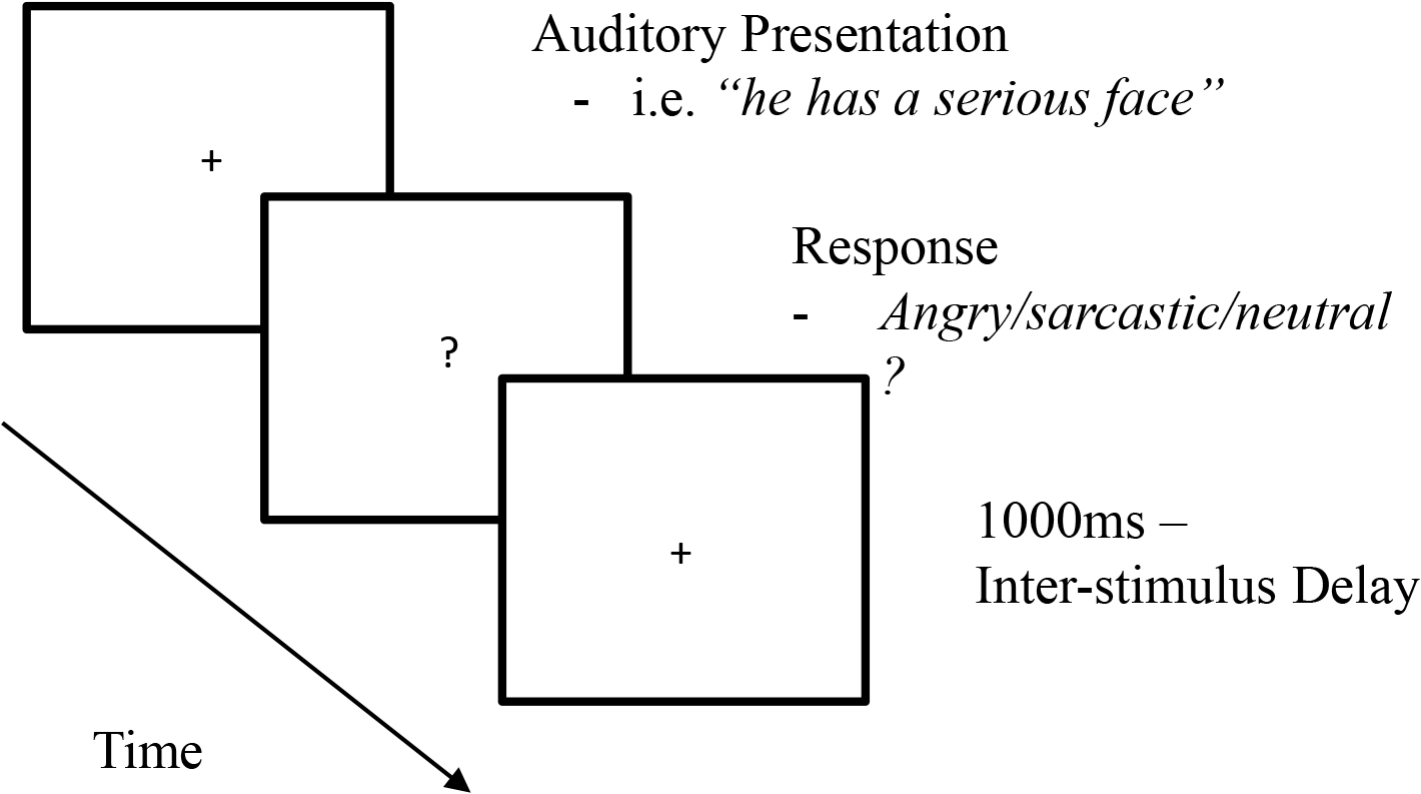
Stimulus presentation for Experiment One.

E-Prime software version 2.0 (Psychology Software Tools, Inc., Pittsburgh, PA) was used for stimulus presentation via a Compumedics NeuroScan Stim AUDIO system using Verbatim headphones. Participants were systematically allocated to one of the three counterbalanced groups. The stimuli were presented in a pseudorandom order. Stimulus triggers were programmed such that both head and body structure were captured for later processing, although only results from the triggers aligned with the body structure are reported in subsequent sections (i.e., the onset of each epoch was aligned with the start of the body and not the head structure). In terms of actual stimuli presentation (see Fig 2), before and during sentence presentation a fixation cross was located centrally. At the termination of each sentence, the fixation cross was replaced with a question mark and only then were participants able to make a response. Following the response, the fixation cross was displayed again after a button-triggered response by the participant. An inter-stimulus delay of 1000ms with a blank screen was present between trials.

#### Procedure

Participants were seated comfortably, given the study information and asked to read and sign informed consent. Before the study commenced, participants were asked to read an initial screen with instructions on it carefully before continuing. Participants were then instructed to pay attention to the speaker’s tone of voice and indicate whether the tone was angry, sarcastic, or neutral by pressing one of three possible keys. Moreover, it was expressed that breaks could be taken, if necessary, at any time where a response was required. In addition, participants were informed about the sensitivity of the EEG to artifacts such as eye movement and muscle activity and were therefore asked to remain as still and relaxed as possible, fixated on the fixation point, and to refrain from excessive blinking, eye, and muscular movements.

#### EEG Recording and Pre-processing

Electroencephalogram (EEG) was obtained with a NeuroScan System SynAmps RT amplifier on a Dell Optiplex 780 computer system. The EEG was located in an electrically shielded room. The EEG was recorded with 64 Ag–AgCl electrodes mounted in an elastic cap (International 10/10 System, NeuroCap) sampling at 500Hz. EEG data was referenced online to the FCz electrode while AFz served as the ground. The EEG signal was recorded online with NeuroScan 4.3 software. The impedance for all the electrode sites was kept below 10kΩ.

The data was pre-processed and analyzed offline using EEGLab software [48] in MATLAB (R2010b, 7.11). Initially, epochs of the data for target body structure stimuli were extracted and a band pass filter was set offline between 1 and 35Hz. The data was then decomposed by Independent Component Analysis (ICA, runica algorithm). Subsequently, components identified by the ICA were studied to remove eye movement artifacts, blinks, cardiac rhythm, and any notable muscular or movement artifacts from the signal. The trace was then visually inspected to reject noisy trials and any trials containing notable artifacts. Across conditions, an average of 9.3% of trials from each participant were rejected by this procedure. EEG epochs (-200–1000ms) were time-locked to the onset of the body structure, baseline corrected (-200–0ms) and re-referenced offline to a common average reference.

#### Data Analysis

**Fig 3:**
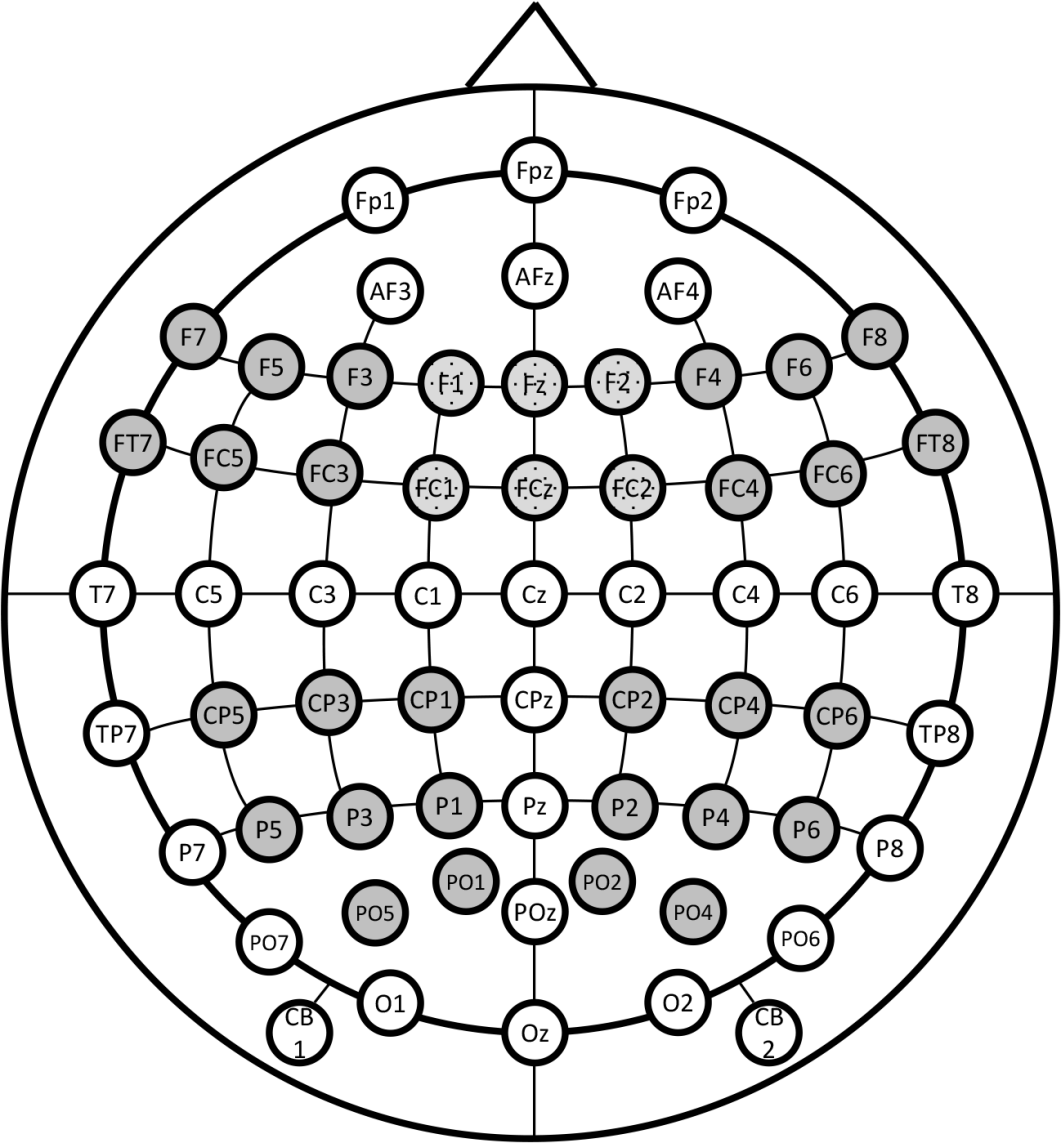
Head-map of electrode positions displaying regions of interest (anterior: left, central, right; centro-parietal: left, right).

According to previous research [25,27,28,49], the following time ranges were chosen to examine various ERP components: 100–200ms, 200–350ms, and 450–700ms. Previous research also demonstrates anterior dominant effects for the early time windows (100-200ms and 200-350ms [25,27,28,31,32,49] and this was corroborated by visual inspection of the effects in the current data. Consequently, the right and left anterior quadrants were statistically examined, as were the fronto-central electrodes above the midline (see Fig 3). Alternatively, late positive effects (>450ms) typically reveal a centro-parietal maximal distribution [25,38,49,50] and hence the left and right centro-parietal quadrants were examined in the 450-700ms time-window (see Fig 3). To examine these, mean amplitudes were calculated across prosodic condition (neutral, attitudinal, emotional) and region (left, centre, right in the two earlier time windows, and left and right hemisphere in the latter one), and these were considered repeated factors in the analysis.

## Results

**Fig 4:**
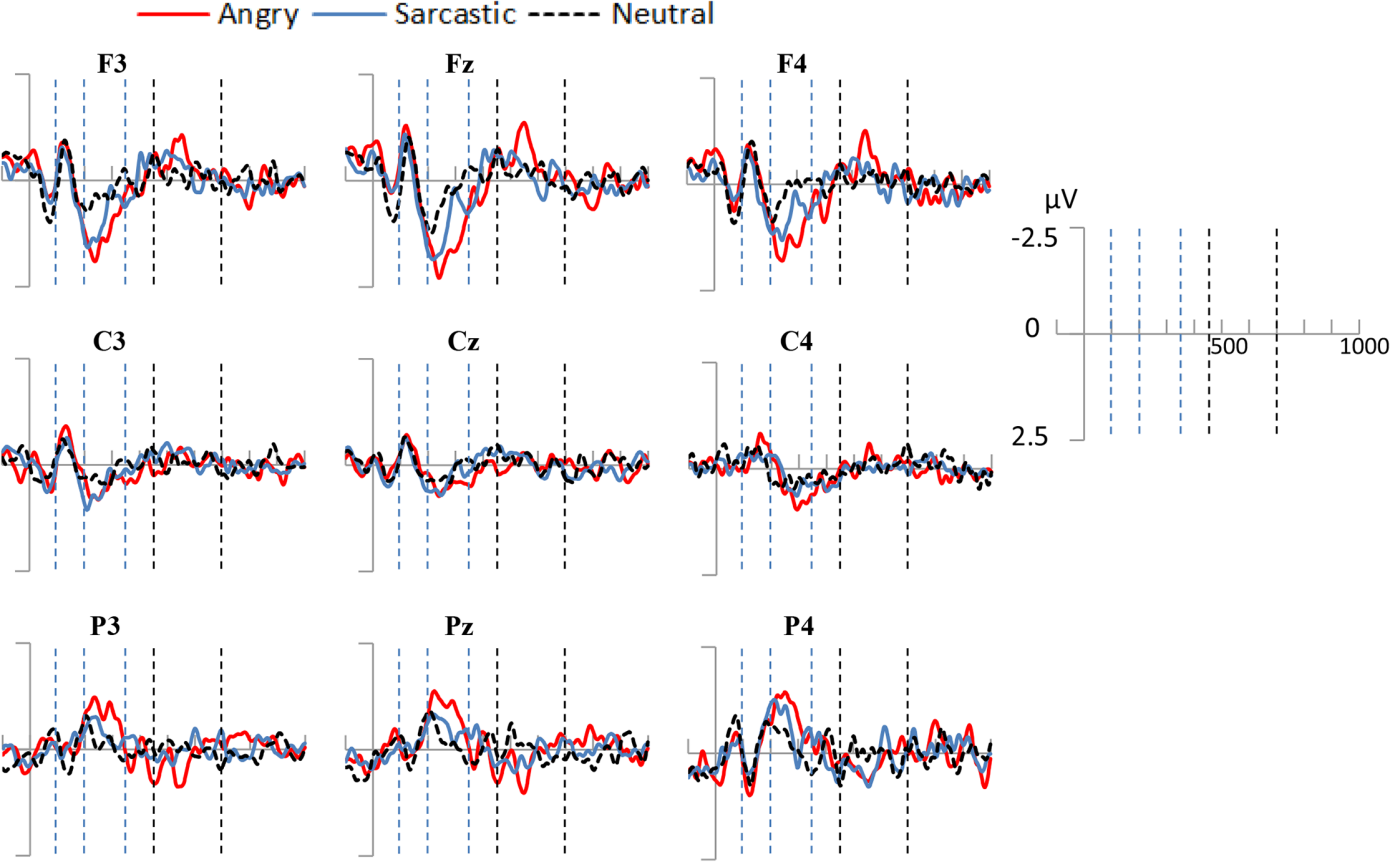
ERPs elicited by angry, sarcastic, and neutral sentences at selected representative electrode-sites in Experiment one. The two areas between the first three dotted lines represent the two earlier time windows examined (100-200ms and 200-350ms). The two latter dotted lines represent the later (450-700ms) time window.

The data was first screened for outliers with respect to grand average amplitude for hemisphere, prosodic condition, and time-window. No outliers were identified with values greater than 3 standard deviations from the mean. Sphericity of the data was also assessed using Mauchley’s test. When this test was violated, a Greenhouse-Geisser correction was used, as is standard with EEG data [51]. A number of representative electrodes are shown in Fig 4. As can be seen, the dominating response and largest difference between the prosodic conditions occurred in the anterior electrodes, with the components in the posterior region showing a mirrored yet markedly reduced effect.

### Early Anterior Negativity (100-200ms)

**Fig 5:**
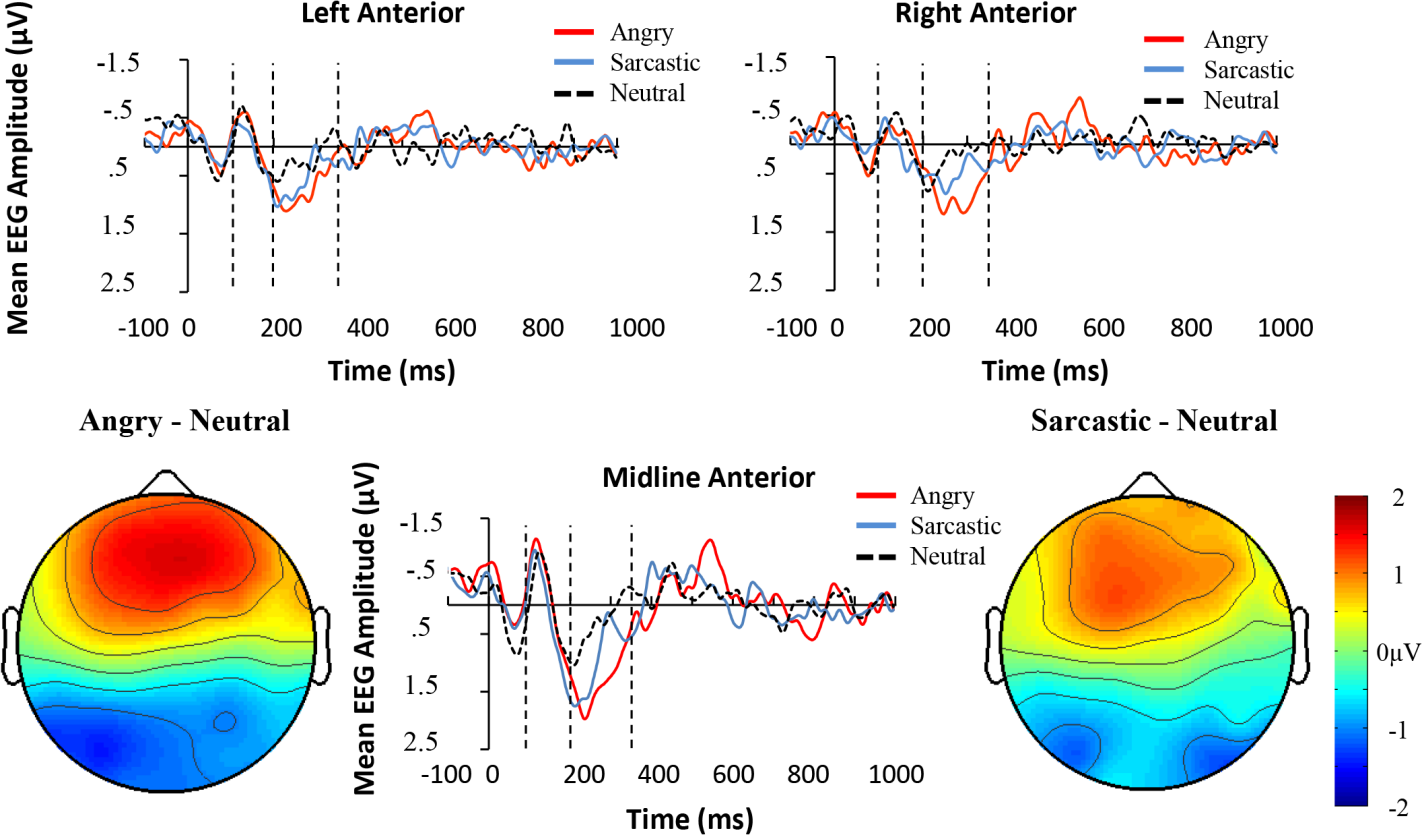
Grand average ERPs for the three conditions averaged over the anterior left (F7, F5, F3, FT7, FC5, FC3), central (F1, FZ, F2, FC1, FCZ, FC2), and right (F8, F6, F4, FT8, FC6, FC4) electrode regions in Experiment one. The space between the dotted vertical lines represents the time frames that the average activation was derived from for the statistical comparisons. The whole-head activation maps are for Angry-Neutral and Sarcastic-Neutral comparisons at 250ms.

A negative peak was observed for all conditions in the 100-200ms time-window, and was examined using a 3 (Prosodic Condition) × 3 (Region) ANOVA. Contrary to expectations, there was no significant effect of mean EEG activity across the prosodic conditions and nor was there a significant effect of hemisphere or interaction between the two, all *F*s < 1 (see Fig 5).

### Early Anterior Positivity (200-350ms)

Visual inspection of the data revealed an anterior positive deflection at 200-350ms. Contrary to expectations, this potential was relatively similar for both angry and sarcastic violations, therefore measures of peak onset latency were not performed. A 3 (Prosodic Condition) × 3 (Region) ANOVA revealed that there was a significant effect of Prosodic Condition (*F*(2, 28) = 9.85, *p* < .005, *partial η^2^* = .41) and Region (*F*(2, 28) = 13.01, *p* < .001, *partial η^2^* = .48), as well as an interaction between the two (*F*(4, 56) = 3.02, *p* < .05, *partial η^2^* = .18) (see Fig 5).

Further planned comparisons were conducted to examine the difference in amplitude deflection in neutral-angry, neutral-sarcastic, and angry-sarcastic comparisons. The results showed that there was a significant difference between the angry and neutral (*F*(1, 14) = 14.61, *p* < .005, *partial η^2^* = .51) and the sarcastic and neutral sentences (*F*(1, 14) = 8.48, *p* < .05, *partial η^2^* = .38), but only a marginal difference between the sarcastic and angry sentences (*F*(1, 14) = 4.37, *p* = .066, *partial η^2^* = .22). There was also a marginal interaction between Region and Prosodic Condition with the angry and neutral sentences (*F*(1, 14) = 3.64, *p* = .077, *partial η^2^* = .21), and a significant interaction with the sarcastic and neutral ones (*F*(1, 14) = 6.73, *p* < .05, *partial η^2^* = .31). This appeared to be caused by higher positive potentials in the fronto-central compared to lateralized regions in the sarcastic and angry condition compared to the neutral one.

### Late Posterior Positivity (450-700ms)

**Fig 6:**
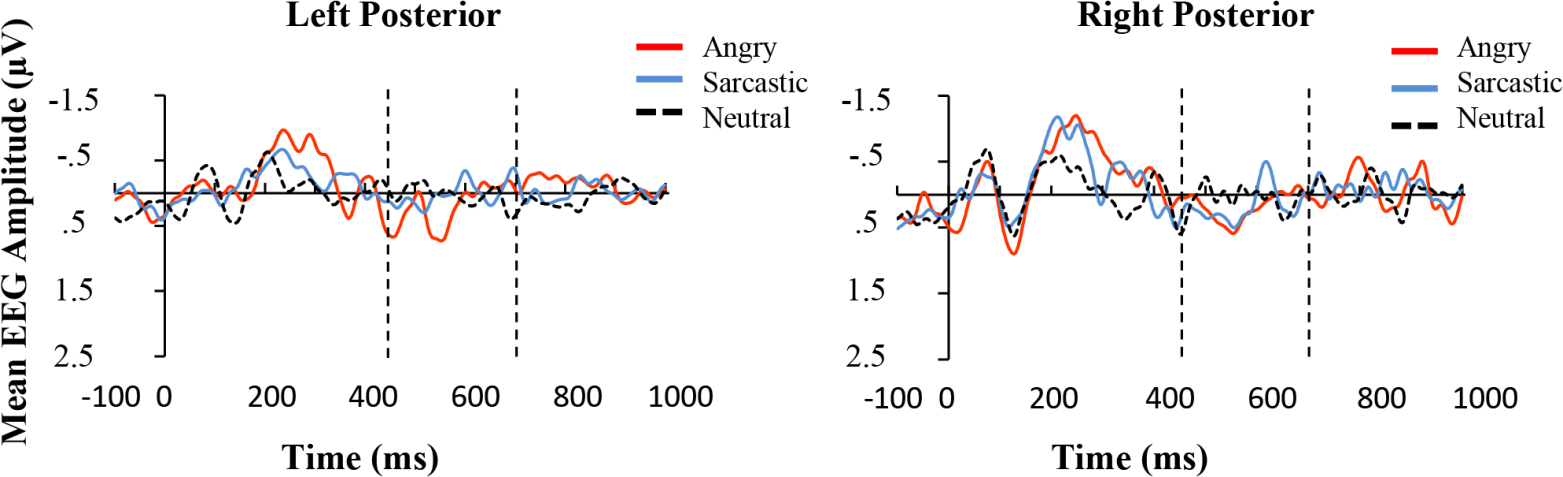
Top Panel: Grand average ERPs for the three conditions averaged over the left (CP5, CP3, CP1, P5, P3, P1, PO5, PO3) and right (CP6, CP4, CP2, P6, P4, P2, PO6, PO4) electrode regions in Experiment one. The space between the dotted vertical lines represents the time frame that the average activation was derived from for the statistical comparisons.

Despite predictions, visual inspection of the data revealed only a slight late occurring positive deflection in the centro-parietal regions in the 450-700ms time-window (see Fig 6). A 3 (Prosodic Condition) × 2 (Hemisphere) ANOVA showed that this difference was not significant across the prosodic conditions (*F*(2, 28) = 1.23, *p* = .31, *partial η^2^* = .081), and nor was there an effect of hemisphere (*F*(1, 14) = 1.08, *p* = .32, *partial η^2^* = .07) or an interaction between the two (*F*(2, 28) = 1.94, *p* = .16, *partial η^2^* = .12)

Overall, the results from Experiment one demonstrate a divergence of mismatching angry and sarcastic violations from neutral sentences at 200-350ms post violation onset. Importantly, the significant positive deflection occurred at a similar time-course for both emotional and attitudinal violations. Alternatively, the results did not reveal any significant differences between the conditions with respect to early negative or late positive potentials.

## Experiment Two

Experiment two was run to examine the effect of task demands on prosodic processing. It was identical to Experiment one except the task was altered so that the main focus was not on prosody. The intention was to examine the extent to which prosodic processing is done automatically and how processing changes with altered task demands. To do this, participants were simply presented sentences and asked to determine if a probe word occurred in them.

### Method

#### Participants

Fifteen healthy individuals (9 males, 6 females), aged 19–34 years (*M =* 23.67; *SD* = 3.66), participated. None had participated in Experiment one. All participants were English speakers with normal or corrected-to-normal vision, without any psychiatric or neuropsychological disorder and were not currently taking any psychiatric drugs or substances, as provided by self-report. All participants provided informed written consent and were compensated for their participation. This experiment was approved by the ethics committee of Swinburne University of Technology.

#### Stimuli and Procedure

Stimuli and procedure were identical to Experiment one except that in this experiment participants were told to “pay attention to the words spoken and when a word is visually presented in the center of the screen, indicate whether the word was present or not in the previous sentence”. In terms of the stimuli presentation, the fixation cross was either followed by an inter-stimulus delay of 1000ms or by the presentation of a single probe word. The probe word was presented, on average, once every six sentences and had a 50% chance of being in the sentence. Words presented were in an 18 point font in the middle of the screen and remained until a button triggered response by the participant.

#### EEG Recording, Pre-processing and Data Analysis

The equipment used and the steps taken during EEG recording, pre-processing, and data analysis were identical to Experiment one. Across conditions, 7.3% of trials were rejected per participant, on average, in pre-processing.

## Results

**Fig 7:**
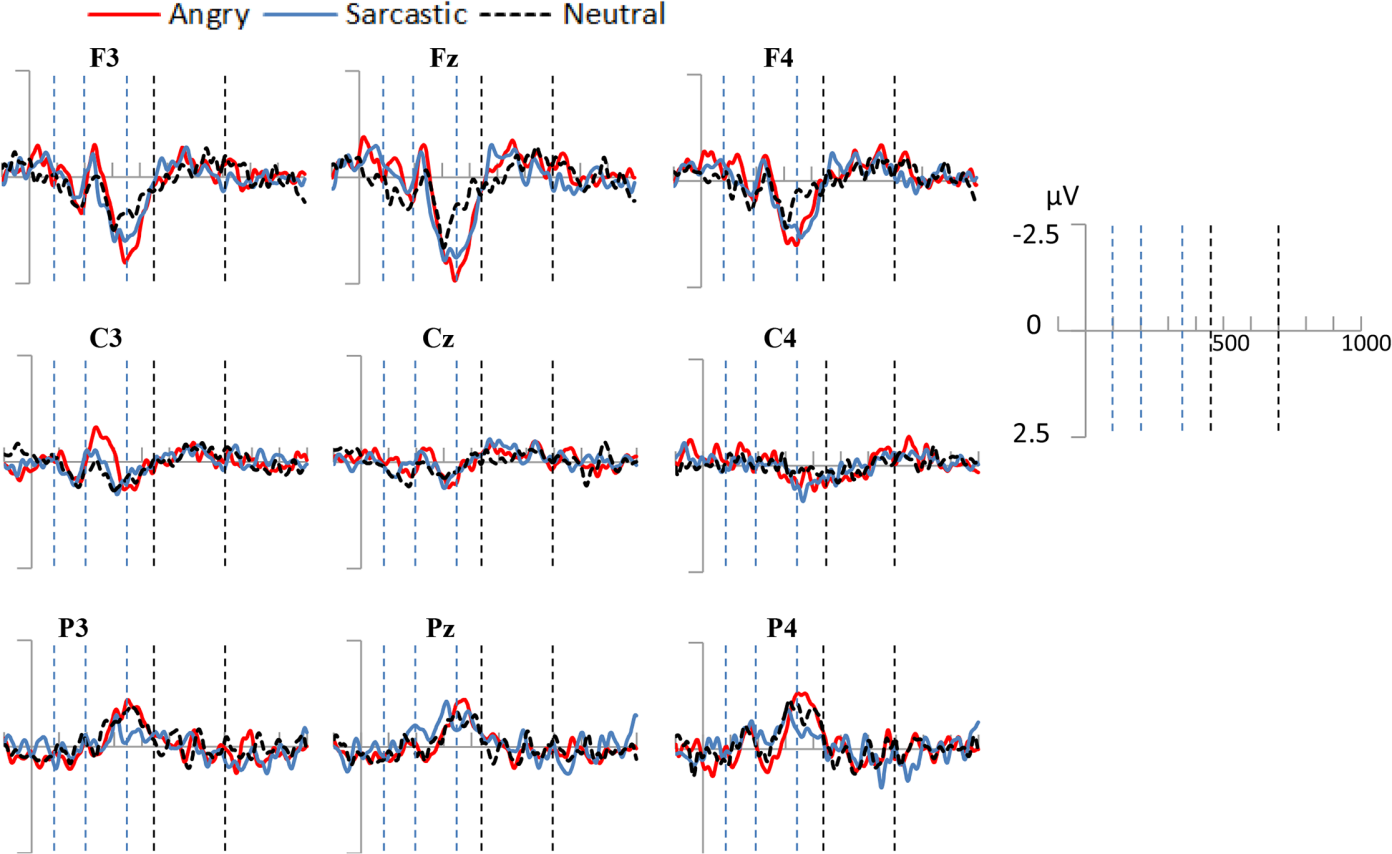
ERPs elicited by angry, sarcastic, and neutral sentences at selected representative electrode-sites in Experiment two. The two areas between the first three dotted lines represent the two earlier time windows examined (100-200ms and 200-350ms). The two latter dotted lines represent the later (450-700ms) time window.

As with Experiment one, the data was first examined for outliers in the three different time windows, and no case revealed a value greater than 3 standard deviations from the mean. A number of representative electrodes appear in Fig 7. As can be seen in that figure, like Experiment one, the largest differences between the conditions occurred in the anterior electrodes, with the components in the posterior region showing a reduced effect.

### Early Negativity (100-200ms)

**Fig 8:**
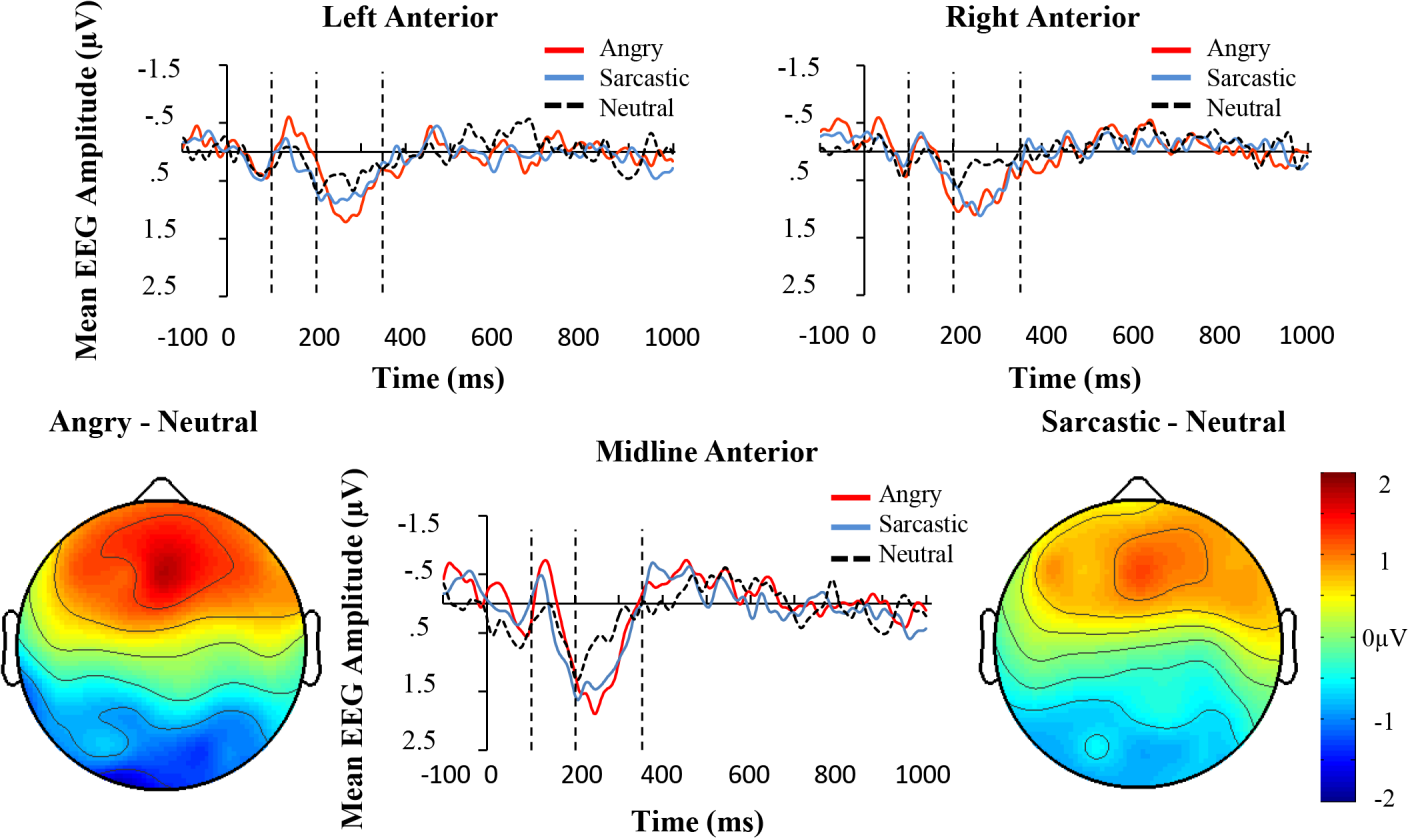
Grand average ERPs for the three conditions averaged over the anterior left (F7, F5, F3, FT7, FC5, FC3), central (F1, FZ, F2, FC1, FCZ, FC2), and right (F8, F6, F4, FT8, FC6, FC4) electrode regions in Experiment two. The space between the dotted vertical lines represents the time frames that the average activation was derived from for the statistical comparisons. The whole-head activation maps are for Angry-Neutral and Sarcastic-Neutral comparisons at 250ms.

Unlike Experiment one, within the early negativity time window (100-200ms), a 3 (Prosodic Condition) × 3 (Region) ANOVA showed that there was main effect of prosodic condition (*F*(2, 28) = 4.41, *p* < .05, *partial η^2^* = .24), although no effect of Region (*F*(1.28, 28) = 2.53, *p* = .12, *partial η^2^* = .15) nor interaction between the two (*F* < 1). Further contrasts showed that the angry sentences displayed a more negative going wave compared to the neutral (*F*(1, 14) = 7.23, *p* < .05, *partial η^2^* = .34) and sarcastic ones (*F*(1, 14) = 9.76, *p* < .001, *partial η^2^* = .41), whereas the sarcastic and neutral sentences did not differ significantly (*F* < 1). The results appear in Fig 8.

### Early Positivity (200-350ms)

Similar to Experiment one, mismatching conditions revealed a positive deflection in the 200-350ms time-window (see Fig 8). In particular, a 3 (Prosodic Condition) × 3 (Region) ANOVA revealed that there was a significant effect of Prosodic Condition (*F*(2, 28) = 6.53, *p* < .001, *partial η^2^* = .32), a significant effect of Region (F(2, 28) = 8.91, p < .005, *partial η^2^* = .39), but no significant interaction (*F*(4, 56) = 1.11, *p* = .36, *partial η^2^* = .074).

Further planned comparisons were conducted to examine the difference in amplitude deflection in neutral-angry, neutral-sarcastic, and angry-sarcastic comparisons. The results showed that there were differences between the angry and neutral (*F*(1, 14) = 13.39, *p* < .005, *partial η^2^* = 48) and the sarcastic and neutral sentences (*F*(1, 14) = 11.32, *p* < .01, *partial η^2^* = .45), but not the sarcastic and angry ones (*F* < 1).

### Late Posterior Positivity (450-700ms)

**Fig 9:**
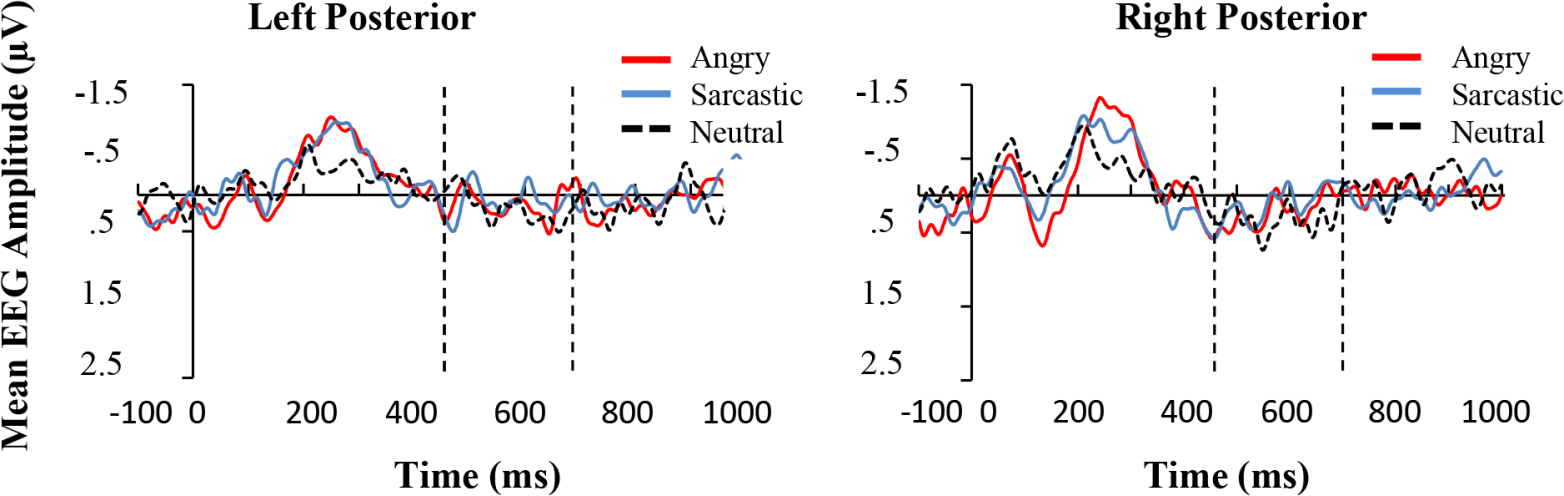
Top Panel: Grand average ERPs for the three conditions averaged over the left (CP5, CP3, CP1, P5, P3, P1, PO5, PO3) and right (CP6, CP4, CP2, P6, P4, P2, PO6, PO4) electrode regions in Experiment two. The space between the dotted vertical lines represents the time frame that the average activation was derived from for the statistical comparisons.

Like the first experiment, a 3 (Prosodic Condition) × 2 (Hemisphere) ANOVA showed no significant effect of Prosodic Condition (*F < 1)*, although there was a significant effect of hemisphere (*F*(1, 14) = 5.98, *p* < .05, *partial η^2^* = .30), with the right hemisphere having a higher mean amplitude than the left. The results appear in Fig 9.

## Summary of Experiment One and Two

**Fig 10:**
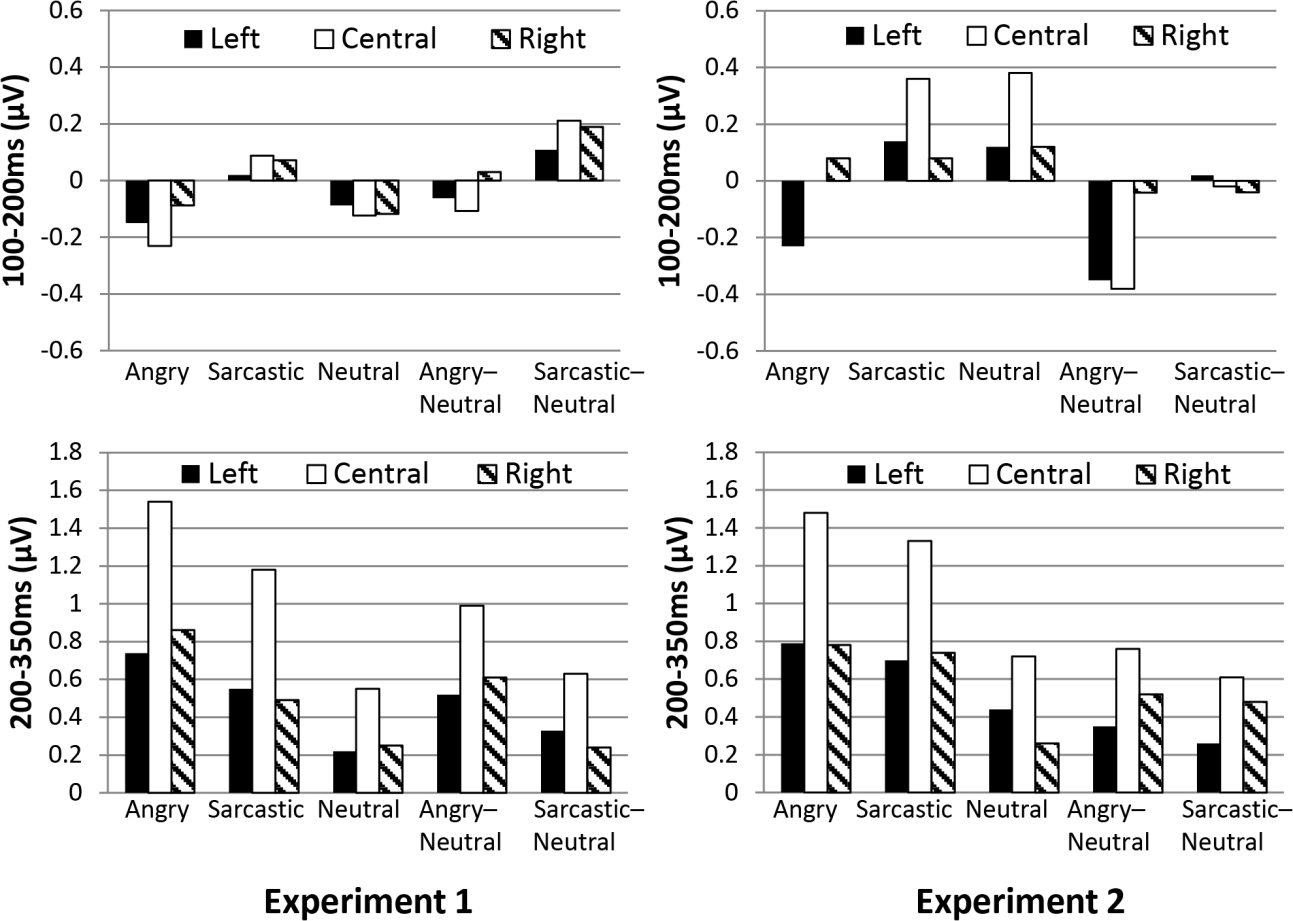
Mean amplitude (μV) of the angry, sarcastic, and neutral sentences as a function of anterior electrode region (Left, Central, and Right) in the two earlier time windows.

Overall, the results of Experiment one and Experiment two principally diverge in relation to the early time-window, where the angry utterances displayed a more negative going wave than either the sarcastic or positive ones and as can be seen, this occurred mainly in the left and fronto-central regions (see Fig 10). In terms of the results 200-350ms post violation, both experiments showed a pattern where there was a divergence between the mismatching angry and sarcastic conditions compared to the neutral controls, with larger differences occurring in the fronto-central compared to lateral regions. This caused a significant interaction in Experiment one but not Experiment two, although as can be seen from the figure, despite this difference, the pattern of results was very similar. To examine this further, an ANOVA was conducted to investigate if there was a 3-way interaction between Experiment (one vs. two), Region, and Prosodic condition. The result was not significant (*F* < 1). This suggests that there were no obvious meaningful differences between the experiments at this time window.

## General Discussion

### Overview of Aims and Findings

The main aim of the present study was to investigate the neural processes underlying non-verbal emotional and attitudinal speech. More specifically, the study examined ERP components in response to violations of prosodic expectancy and the extent to which the neural processes for angry prosodic violations differed from sarcastic prosodic violations. Overall, the results revealed that mismatching emotional and attitudinal prosodic expectancy violations diverge from matching neutral sentences at 200-350ms post-violation onset; eliciting an anterior distributed positivity. Contrary to expectations, the positivity reported for attitudinal prosodic violations did not appear to occur later in time compared to emotional violations. Also inconsistent with expectations, no significant late positive complex effects were found. There were, however, minor distributional differences between the two types of prosody in the two experiments. In particular, in Experiment one where prosody was in task focus, no significant differences between prosodic conditions were found with the early negative effect, whereas in Experiment two they were.

### The Early Negative Effect (100-200ms)

Whilst a negative component was revealed for all conditions at 100-200ms, a significant difference between prosodic conditions was only found in the second experiment, where angry utterances displayed a more negative-going wave than the neutral or sarcastic ones. This result is similar to other studies showing emotional deviations elicit early anterior dominant negative potentials in both oddball paradigms [31,52] and PEPs [25], with the results being more left lateralized in our study. Typically, these potentials are argued to reflect early mechanisms of physical change-detection, including differences in sound (i.e., mismatch negativity [53]; MMN). As the paradigm adopted in the current study did not reflect a typical oddball paradigm, it is somewhat surprising that that these results were observed. However, Chen et al. [25] reported a significant anterior negative component for emotional prosodic violations that occurred independent of task demands and over and above spectrally rotated counterparts (i.e., it occurred after controlling for the basic acoustic features of the words while subtracting the emotional significance), which was, in turn, likened to mismatch negativity. One reason that we may have failed to find the effect in Experiment one could be due to different processing demands of the tasks. Alternatively, it could simply be a power issue. In particular, the effect may be relatively small considering the neutral vs. angry stimuli used by Chen et al. were only significant at the *p* < .05 level in their first experiment using prosodic classification and the effect was not found by Kotz and Paulmann [27] at all. In addition, Schirmer et al. [31] revealed significant MMN only for women. Given that the current study had a relatively small sample with a greater male to female ratio, it is thus possible that early negative effects were not elucidated consistently through grand averages. Finally, the optimum number of stimuli needed per cell to consistently find such an early effect is likely to be much greater than this study and many of the other studies used [35].

### The Early Positive Effect (200-350ms)

#### Emotional Prosody

As anticipated, emotional prosodic deviations resulted in a significant positivity, which occurred between 200-350ms post violation onset. This finding indicates that mismatching emotional prosody is detected at an early stage of processing. Consistent with predictions, this finding is comparable to the PEP effects reported for angry emotional deviations occurring in a similar time window [25,27]. It is similarly consistent with the salience hypothesis [29], where prosodic deviations related to emotional content are identified due to the saliency of specific acoustic cue patterns in emotionally laden stimuli.

An alternative view of the positivity can be made with respect to a P200 component, which is also consistent in terms of the distribution, morphology, and latency. In particular, the PEP effects reported by Kotz and Paulmann [27], Paulmann and Kotz [28] and Paulmann et al. [29] exhibited a slightly later onset (~350ms) and a more sustained potential. As such, it is possible that the PEP effect reported by Chen et al. [25] is in fact a P200 potential. The P200 has also been found in studies employing emotional non-verbal vocalizations [32], simple sentences [33], and pseudo-sentences [49]. Furthermore, the P200 is argued to reflect attentional mechanisms of rapid salience detection [49]. While the paradigm used in the current study supports a PEP interpretation, both the PEP and P200 appear to reflect similar processes, that being a detection mechanism which is sensitive to the salience of acoustic parameters. Accordingly, either interpretation provides insight into when and where the type of prosody is detected and processed and thus allocated neural resources. The precise stage or level at which this information is processed, however, remains to be elucidated.

Apart from when the deflection occurred, the topographic distribution of the results was also of interest, with the positivity maximal in anterior regions; consistent with previous literature [25,27,28,32,33,49]. In accordance with Chen et al. [25], the positivity displayed a bilateral anterior distribution, which is also found in neuroimaging data [17,18]. Alternatively, there have been previous reports of right lateralized [27,28] and centro-parietal [29] emotional PEP effects. One possible reason for some of the differences is that, like Chen et al. [25], the stimuli used in this study differed on numerous acoustic cues (i.e., pitch, pitch variability, intensity, intensity range and duration), as they do in normal speech. Alternatively, the stimuli in Kotz and Paulmann [27], and Paulmann et al. [29] differed only in relation to pitch.

The results are also explicable within the cue-dependent hypothesis where the distribution and lateralization of prosodic processing is dependent on the constituent acoustic cues [19]. Within this framework, the right hemisphere processes acoustic information with a low temporal resolution whilst the left hemisphere operates on a higher temporal resolution [20]. Given that research indicates that the right hemisphere may be more specialized for pitch processing [21,29], this may account for the distributional differences for emotional violations between the studies since studies manipulating only pitch would be more likely to elicit mainly right hemisphere differences. The cue dependent hypothesis account does not, however, offer an explanation for centro-parietal emotional deviancy effects reported by Paulmann et al. [29] that were not found in this study.

#### Attitudinal Prosody

Despite the fact that attitudinal prosody had not been previously examined in an expectancy paradigm, it was speculated that prosodic re-analysis processes would occur later in time compared to emotional prosodic violations. Foremost, this hypothesis was proposed in light of the temporal dynamics regarding positive going prosodic ERP potentials and the salience hypothesis of Paulmann et al. [29] such that attitudinal prosody may possess less salient acoustic cue patterns due to conceptual differences between attitudinal and emotional functions. As anticipated, attitudinal/sarcastic prosodic violations elicited a significant positive going deflection. However, contrary to expectations, it occurred in the same time-window as emotional violations (i.e., 200-350ms). Assuming that the PEP effect reflects salience of the stimuli, these results suggest that the angry and sarcastic stimuli used in the current study did not differ significantly in terms of their perceived salience. This explanation is similar for a P200 interpretation of the data, in that if they did differ in terms of salience, the P200 would have showed similar differences as a result, as has been reported when people distinguish between ironic compared to literal sentences [37,50]. This finding thus provides evidence supporting processing similarities for emotional and attitudinal prosody.

### On the Absence of Late Positive Effects

As noted in the introduction, we expected to find late positive potentials during prosodic evaluations (i.e., prosodic classification) due to online integration processes. Such components around and over 600ms have been found in studies of emotional prosody [25,49], linguistic prosody [24,26,29] and attitudinal prosody [37,38]. These were not, however, found in this study. Given that late positive effects are most commonly argued to reflect deeper semantic and linguistic re-integration processes, one explanation for the lack of late positivity effects may be that the stimuli used in the current study were not processed in enough depth. In turn, the departures from neutral to emotional or attitudinal prosody may not have required contextual re-integration. This is plausible considering that the previous studies demonstrating late positive effects provided greater contextual cues. For instance, the head structures used in Chen et al. [25] were more complex and the paradigms used in Regel et al. [37] and Rigoulot et al. [38] were inherently contextual (i.e., discourse contexts and question-answer dialogue, respectively).

### Effect of Task Demands

Primarily, the results suggest that very early effects might be modulated by task demands, with angry sentences producing a more negative going wave between 100-200m in the second but not first experiment. Interestingly, the same result was not found with the sarcastic sentences. This may have been because early acoustic differences between the neutral and angry sentences were greater than the sarcastic and neutral sentences on the measures examined, and is thus still consistent with previous findings showing that the N100 can be modulated by selective attention mechanisms [54]. Alternatively, as noted above, this difference may also simply be a power issue.

In terms of the results from the second time window (200-350ms), there was very weak evidence that the prosodic processing of sarcasm and anger might be dependent on task demands. In particular, in the first experiment, there was a marginal difference between the angry and sarcastic sentences, but in the second experiment, there was no significant difference. Given the weakness of this result and the very similar pattern of results across the two experiments, it seems too early to attribute anything meaningful to this finding. In terms of the late time window (450-700ms), we had expected to find differences where sarcasm was more affected by task demands than anger, as would be predicted given the processes hypothesized to occur in the third stage of prosodic processing by Mitchell and Ross [2]. However, since neither the angry nor sarcastic condition showed significant positive effects in that time window, it is not surprising that we did not find any differences.

Despite not finding important differences with the PEP effect between tasks in our study, there were some differences between the current and previous results. In particular, Chen et al. [25] and Kotz and Paulmann [27] performed similar task demand manipulations where classification and probe-verification tasks were used, but their experiments produced different distributional effects. Notably, however, each study also demonstrated altered distributional effects across tasks. Chen et al. [25] showed a comparable anterior bilateral PEP effect during the prosodic classification task, whereas the reverse pattern was found for the probe verification task; a mid-left lateralized effect. Alternatively, Kotz and Paulmann [27] revealed a whole-head bilateral PEP effect for a probe-verification task and a right lateralized fronto-central effect for a prosodic classification task. These results suggest that the positivity clearly seems to be sensitive to distributional changes to which task demands appear to be influential. This may suggest that the neural resources responsive to the saliency of acoustic cue patterns are not fixed, but rather dependent on where attention is allocated [18]. Moreover, aspects of the tasks that cause distributional differences are currently unclear, partially due to the fact that the different types of tasks may load quite differently on different cognitive processes. For example, with prosodic classification, it is plausible that one could stop focusing on the task as soon as a change is detected whereas with probe verification, one is obliged to commit the entire sentence to memory because the probe word appears after the sentence has completed. Thus the way memory works, which can be affected quantitatively and qualitatively by emotional stimuli (e.g., [55]), differs in the two tasks, and it is therefore certainly not inconceivable that this could lead to different distributional effects including interactions with different prosodic conditions. Further differences could emerge from language differences if, for example, the automaticity at which cues are processed across languages differ. Speakers of tonal languages, like Chinese, might, for example, process pitch information differently compared to speakers of non-tonal languages like English [56,57].

### Limitations

Foremost, a principal shortcoming of the current experiments was the comparatively small number of participants used. This means that the study may have lacked power, especially in the early time window where either null or relatively weak effects have been reported. However, despite this consideration, there are important results which would be unlikely to change with additional participants. In particular, with our angry utterances, there was almost no lateralization of the results, as was also found by Chen et al. [25], and this was unlike Kotz and Paulmann [27] who found right lateralization using the same task. Unless our participants were especially atypical, it would be very surprising if testing additional ones would not only dilute the original pattern but also then cause a right lateralized result. This suggests that there are likely to have been significant variances between tasks, stimuli, or sample populations that cause important differences, despite the number of participants.

Another limitation is that the sarcastic stimuli used were constrained by their selection, where only stimuli that were obviously of a sarcastic intonation were used. This was necessary because they were used out of context, all were sarcastic based purely on intonation, and we wanted our participants to be able to classify them with high accuracy. As a result, it is likely that they were relatively homogenous compared to more natural sarcastic prose, and that they were also easy to recognize based on their phonetic characteristics. These factors may have biased participants towards processing mainly the phonetic characteristics of the sentences rather than the meaning and may be one reason for the lack of late positive effects. A related limitation was that the current study did not systematically control the extent to which the sarcastic and angry stimuli differed on phonetic characteristics. In terms of the phonetic variables examined, the angry stimuli departed more from the neutral stimuli than the sarcastic stimuli. This included a very large deviation in fundamental frequency with the angry compared to the sarcastic stimuli. As a result, it is possible that participants could have potentially differentiated all three types of stimuli based on simple phonetic characteristics. Such differences could have led to task-specific effects where participants were biased towards using a simple phonetic processing strategy classification strategy when a violation occurred compared to processing the actual emotional and attitudinal meanings of the sentences. Thus, the minor differences elucidated between sarcastic and attitudinal prosody could be related to strategy choice, and conversely, differences related to the deeper processing of sarcastic attitudes could potentially have been concealed. Future research manipulating characteristics of the stimuli or using different types of task (e.g., a probe question concerning the meaning of the sentence) may help elucidate the extent to which the strategy affects the results.

## Conclusion

The present study aimed to examine the neural processes underlying emotional and attitudinal prosody; operationalizing emotional prosody as an angry intonation and attitudinal prosody as a sarcastic intonation. Overall, the results indicate that angry and sarcastic violations appear to be processed over a similar temporal course and that their cortical distributions are also quite similar, with meaningful task related differences only found very early in processing. These results add to the literature on emotional prosodic processing, replicating early anterior positive processing effects. This study is also novel in trying to disentangle the processing of affective prosody into its constituent emotional and attitudinal counterparts, in addition to providing insight into the neural underpinnings of acoustically driven attitudinal prosody.
